# The Usage of Different Hyaluronic-Acid-Containing Artificial Tears and the Treatment Outcome of Intense Pulsed Light Therapy for Dry Eye Disease: A Retrospective Cohort Study

**DOI:** 10.3390/diagnostics14161796

**Published:** 2024-08-16

**Authors:** Chia-Yi Lee, Shun-Fa Yang, Yun-Chen Chen, Chao-Kai Chang

**Affiliations:** 1 Institute of Medicine, Chung Shan Medical University, Taichung 40201, Taiwan; 2 Nobel Eye Institute, Taipei 10041, Taiwan; 3 Department of Ophthalmology, Jen-Ai Hospital Dali Branch, Taichung 41265, Taiwan; 4 Department of Medical Research, Chung Shan Medical University Hospital, Taichung 40201, Taiwan; 5 Chaomuhean Clinic, Taipei 10689, Taiwan; 6 Department of Optometry, Da-Yeh University, Chunghua 51591, Taiwan

**Keywords:** dry eye disease, intense pulsed light, tear break-up time, hyaluronic acid, Schirmer test

## Abstract

In this study, we aimed to investigate the effect of different hyaluronic acid (HA)-containing artificial tears on dry eye disease (DED) treatment in combination with intense pulsed light (IPL) therapy. A retrospective cohort study was conducted, and the participants received IPL therapy and HA-containing artificial tears. There were 42 and 40 eyes in the 0.10% and 0.15% HA groups after selection, respectively. The main outcomes were the postoperative non-invasive tear break-up time (NITBUT), Schirmer II test results, ocular surface stain, and numbers of DED-related symptoms. A generalized linear model was utilized to produce the adjusted odds ratio (aOR) and 95% confidence interval (CI) of the main outcomes between groups. At the three-month follow-up, the NITBUT was significantly higher in the 0.15% HA group (*p* = 0.023), and the NITBUT recovery was also significantly better in the 0.15% HA group compared to the 0.10% HA group (*p* = 0.039). The multiple DED-related symptoms significantly correlated with no DED symptom improvement in both the 0.10% and 0.15% HA groups (both *p* < 0.05), while the low pre-treatment NITBUT was marginally related to no DED symptom improvement in the 0.10% HA group (*p* = 0.047). A low NITBUT and Schirmer II test result correlated with no DED symptom improvement in both the groups (all *p* < 0.05). In conclusion, the application of 0.10% and 0.15% HA-containing artificial tears revealed similar effects to IPL therapy for DED.

## 1. Introduction

Dry eye disease (DED) is a common eye disease that comprises mucin deficiency, evaporative excess, aqueous deficiency, and the mixed type [1]. A prevalence of approximately 20 percent was reported in the Asian and Pacific population [2]. Dryness, discharge, a foreign body sensation, grittiness, photophobia, burning, and redness are common presentations related to DED [3]. Severe and refractory DED may interfere with visual acuity and quality of life [4], and keratitis could occur in these cases [5].

Certain medical and surgical therapies have been used to treat DED [6]. Artificial tears have been utilized for simple and postoperative DED and are, by far, the most common therapy [7,8]. Topical steroids are another eyedrop that has been used to manage DED with acceptable effect. The administration of both artificial tears and topical steroids was able to more effectively reduce the severity of DED but without severe adverse effects [9]. In relentless cases of DED, surgery involving amnion membrane transplantation may be performed [10]. In recent years, hyaluronic-acid-containing artificial tears have been widely applied in clinical practice because of their promising treatment outcomes [11,12,13].

Intense pulsed light (IPL) therapy has been utilized to relieve DED symptoms for decades [14]. The thermal function of IPL therapy was shown to be competent in efficiently controlling DED, and this effect is even better for the evaporative excess type [15,16,17]. Moreover, IPL utilization showed effectiveness in post-keratorefractive surgery DED according to preceding publications [18,19,20]. Nevertheless, there is insufficient research that interrogates the adaptability of IPL therapy with different artificial tears. Because artificial tears have different functions depending on their composition, there may be an ideal choice of artificial tear for use with IPL, which remains to be elucidated.

Therefore, the focus of the present study is to assess the therapeutic outcomes of IPL therapy with different hyaluronic acid (HA)-containing artificial tears. The effects of different artificial tears, combined with IPL therapy, on each DED parameter are also surveyed.

## 2. Materials and Methods

### 2.1. Participant Selection

A retrospective cohort study was performed at the Nobel Eye Institute, which is an ophthalmic bloc that comprises more than 15 clinics in the whole Taiwan region. The participants were included if they conformed to the following criteria: (1) age range from 20 to 100 years old, (2) diagnosed with DED at the Nobel Eye Institute, (3) received an automatic ocular surface analyzer test at the Nobel Eye Institute, (4) received IPL therapy at least three times at the Nobel Eye Institute, and (5) visited the Nobel Eye Institute for IPL therapy for at least six months. On the contrary, the following exclusion criteria were adopted to remove participants with a debilitated ocular surface: (1) prior central corneal opacity; (2) prior corneal ulcer; (3) prior central corneal erosion; (4) prior deep corneal infiltration; (5) prior corneal perforation; (6) prior chemical burn that involved the central cornea; (7) prior cicatricial conjunctivitis; (8) prior keratorefractive surgery; (9) prior systemic inflammatory disease including but not limit to rheumatic arthritis, Sjogren syndrome, diabetes, systemic lupus erythematous, and thyroid eye disease; and (10) used more than one type of artificial tear during the post-treatment period. After the identification of patients from medical records according to the inclusion and exclusion criteria, they were further divided into different groups according to the HA artificial tears they had used. The usage of 0.10% or 0.15% HA artificial tears was decided by our participants because both of these are self-paid in Taiwan. Furthermore, the right eye of each DED participant was included in the present study. After our selection, a total of 42 and 40 eyes were enrolled in the 0.10% and 0.15% HA groups, respectively.

### 2.2. Intense Pulsed Light Therapy

All the IPL therapies were provided by a DED specialist (Y.-C.C.). Local anesthesia gel was administered to the upper eyelid area, lower eyelid area, and upper nasal area, and the participants laid down for about three minutes. A protective shield was installed onto the cornea before the outset of IPL therapy. Then, an 8 × 15 mm square IPL probe was placed on the right upper eyelid area first, and then IPL pulses were applied 10 times with an energy of 15 J/cm^2^; the same energy was applied to the right lower eyelid for another 10 pulses. After the right eyelid was treated, an identical therapeutic protocol was applied to the left eyelid, and then the IPL therapy was performed again for both eyelids. After the whole course of IPL therapy, the participants were guided to a slit-lamp biomicroscope and the physician squeezed their eyelids to clear away the meibomian components. Finally, artificial tears, topical fluorometholone, and carbomer eye ointment were applied to the eyelid and ocular surface.

### 2.3. Dry Eye Evaluation

All the participants in the present study received routine pre-IPL and post-IPL exams. Any previous ophthalmic diseases and surgeries were recorded. The pre-treatment DED exams involved un-corrected visual acuity (UDVA), manifest refraction of both sphere power and cylinder power using an autorefractor (KR-8900, Topcon, Itabashi-ku, Tokyo, Japan), and intraocular pressure (IOP) via pneumatic tonometry (NT-530, NIDEK, Gamagori, Aichi, Japan). Cycloplegia refraction was illustrated as a spherical equivalent (SE) in the present study, which means sphere power plus half of cylinder power. In terms of the DED-related components, non-invasive tear break-up time (NITBUT), eyelid closure rate, lipid thickness, meibomian gland loss rate, and tear meniscus height (TMH) were measured using an automatic ocular surface analyzer (Sbm Device, SBM Sistemi, Strada Torino, Orbassano, Italy). In addition, a Schirmer II test and fluorescein ocular surface stain were performed in all participants. The degree of ocular surface stain was determined according to the Oxford Scheme. The subjective DED symptoms of dryness, itching, burning sensation, grittiness, soreness, fatigue, foreign body sensation, photophobia, redness, and discharge were taken from the patients’ medical documents. The post-treatment examinations of IPL therapy included the UDVA, the IOP, and manifest refraction using the same devices. Furthermore, the fluorescein stain, NITBUT, Schirmer II test, and subjective DED-related symptoms (where relevant) were examined after the IPL therapy. DED-related examinations before and until 3 months after the IPL therapy were analyzed in the present study.

### 2.4. Statistical Analysis

SPSS version 20.0 (SPSS Inc., Chicago, IL, USA) was used to perform all statistical analyses. The statistical power of this study was 0.78 with a 0.05 alpha value, and the medium effect size was generated via G*power version 3.1.9.2 (Heinrich Heine Universität at Düsseldorf, Germany). The Shapiro–Wilk test was performed to survey the normality of these data, and the results displayed non-normal distributions of all data (all *p* < 0.05). A descriptive analysis was performed for the baseline features of the 0.10% and 0.15% HA groups, and the Mann–Whitney U and Chi-squared tests were carried out to analyze the pre-treatment indexes and post-treatment outcomes between the two groups. The Wilcoxon signed-rank test and Chi-squared test were carried out to compare the pre- and post-treatment values of DED parameters to assess their improvements in the 0.10% and 0.15% HA groups. Following this, a generalized linear model was utilized to compare the effect of combined treatment between the two groups regarding the changes in the NITBUT, DED-related symptoms, ocular surface stain, and Schirmer II test results. The adjusted odds ratio (aOR) and related 95% confidence interval (CI) were produced after adjusting for age, sex, manifest SE, and the usage of antidepressants in the multivariable analysis. For the age-based analysis, the study population was divided into those aged 20–60 and those aged 60–100 years old, and the generalized linear model was utilized to evaluate the changes in DED-related parameters between the 0.10% and 0.15% HA groups in different age intervals. In the next step, we separated the participants into those without sign improvement and without symptom improvements. The participants without symptom improvement were defined as no relief of at least one DED-related symptoms three months after the combined treatment. The participants without symptom improvement were defined by the following: (1) an improvement in the Schirmer II test of lower than 2 mm, (2) an improvement in the NITBUT of lower than 2 s, or (3) a decrement in the ocular surface stain score of less than 1 degree. The generalized linear model was utilized again to evaluate the pre-treatment risk factors of no sign/symptom improvements in the two groups. Statistical significance was set at *p* < 0.05 and a *p* value lower than 0.001 was delineated as *p* < 0.001.

## 3. Results

The baseline characteristics of the two groups are demonstrated in Table 1. The mean ages were 46.23 ± 11.72 and 45.96 ± 12.51 in the 0.10% and 0.15% HA groups, respectively, which were without significant difference (*p* = 0.905). Moreover, sex, previous ophthalmic disease, and previous ophthalmic surgery illustrated similar distributions between the two groups (all *p* > 0.05). All the pre-treatment ophthalmic indexes demonstrated similar values between the A and B groups (all *p* > 0.05) (Table 1).

**Figure 1:**
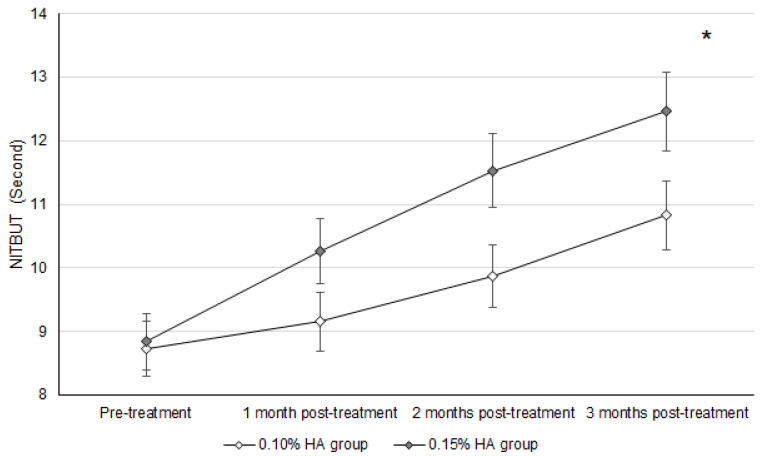
The trend of non-invasive tear break-up times between the two groups. HA: hyaluronic acid; NITBUT: non-invasive tear break-up time. * denotes significant difference between the two groups. A better NITBUT recovery was found in the 0.15% HA group.

**Figure 2:**
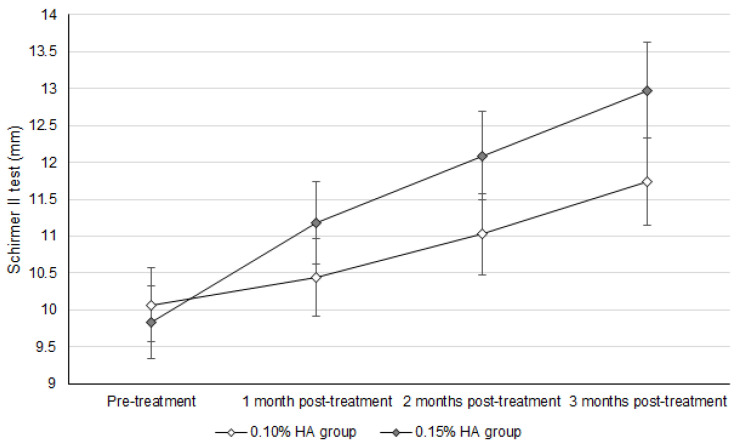
The trend of Schirmer II test results between the two groups. HA: hyaluronic acid. Similar improvements in the Schirmer II test result were found in the two groups.

At the three-month follow-up, the Schirmer II test values, NITBUT, ocular surface stain, and DED-related syndromes were significantly improved after treatment in both the 0.10% and 0.15% HA groups (all *p* < 0.05). For the inter-group analysis, the UDVA, IOP, and manifest SE did not show a significant difference between the two groups (all *p* > 0.05). For the DED-related indexes, the NITBUT was significantly higher in the 0.15% HA group than the 0.10% HA group (*p* = 0.023) (Table 2), while the Schirmer II test results were non-significantly higher in the 0.15% HA group compared to the 0.10% HA group (*p* = 0.101) (Table 2). Concerning the change in DED-related indexes between the two groups, the NITBUT recovery was significantly better in the 0.15% HA group compared to the 0.10% HA group (aOR: 1.143; 95% CI: 0.999–1.256; *p* = 0.039) (Table 3), and the Schirmer II test results also showed a marginally better recovery in the 0.15% HA group compared to the 0.10% HA group (aOR: 1.233; 95% CI: 1.016–1.374; *p* = 0.052) (Table 3). The ocular surface stain and DED-related symptoms did not show significant differences between the two groups (both *p* > 0.05) (Table 3). The trends of improvements in the NITBUT and Schirmer II test results between the two groups are presented in Figure 1 and Figure 2, respectively. The results of the age-based trend analysis showed that the NITBUT has significantly better improvement in the 0.15% HA population than the 0.1% HA population in both the 20–60 (aOR: 1.205; 95% CI: 1.002–1.323; *p* = 0.046) and 60–100 (aOR: 1.289; 95% CI: 1.037–1.465; *p* = 0.022) age subgroups. Moreover, the degrees of improvement in the Schirmer II test results, ocular surface stain, and DED-related symptoms were statistically insignificant between the 0.15% and 0.1% HA populations in both age subgroups (all *p* > 0.05). Furthermore, the usage of antidepressants did not correlate with better improvements in the NITBUT, Schirmer II test results, ocular surface stain, or DED-related symptoms after the IPL treatment (all *p* > 0.05).

Concerning the correlation between pre-treatment indexes and an absence of DED symptom improvements, the multiple DED-related symptoms significantly correlated with no DED symptom improvements in either the 0.10% or 0.15% HA groups (both *p* < 0.05), while the low pre-treatment NITBUT was marginally related to no DED symptom improvements in the 0.10% HA group (*p* = 0.048). The low NITBUT and low Schirmer II test results correlated with no DED sign improvements in either the 0.10% or 0.15% HA groups (all *p* < 0.05).

## 4. Discussion

In the present study, the post-treatment NITBUT was significantly higher in the 0.15% HA group than the 0.10% HA group. On the other hand, the trend of NITBUT improvement was also better in the 0.15% HA group than the 0.10% HA group. Additionally, the predictors for worse post-treatment signs and symptoms were very similar in the 0.15% and 0.10% HA groups.

The reported pathophysiologies of DED’s development vary in existing studies [11,21]. Firstly, ocular surface degradation could lead to its development [22]. Goblet cell deficiency due to autoimmune disorders and chemical burns may impair mucin secretion and cause the mucin-deficiency form [21], which is associated with a prominent DED presentation that includes a harmed corneal epithelium, shortened tear film stability, and irritating DED-related symptoms [5,11]. Furthermore, inflammatory cytokines can induce DED thus increasing interleukin concentration [23]. In detail, the concentration of both tumor necrosis factor family and interferon would be elevated in DED [24]. On the other hand, a decline in ocular surface homeostasis and restricted tear film stability could increase the possibility of DED [1]. A decline in tear film stability is associated with corneal epithelium damage, inducing the inflammatory cytokine release, and then raising the osmolarity of tear film, finally generating an unstable tear film; this is called the vicious cycle of DED development [25]. In addition to the above etiologies, oxidative stress is a valuable component of DED [26]. In a previous article, high oxidative stress was reported to damage the DNA structure and cause lipid peroxidation and a successive DED episode [27]. In another report, the levels of antioxidants would prominently decline in people who received refractive surgery and were diagnosed with prior DED [28]. HA is a long-chain molecule that has demonstrated anti-inflammatory function [13], through which interleukin expression decreases after the utilization of HA and epigallocatechin gallate [29]. Furthermore, large-molecule HA represents a good free radical scavenger and was shown to vitiate oxidative stress in previous research [30]. In addition, the application of HA benefitted corneal re-epithelialization in the mouse cornea [11]. Because HA has several features that can negatively affect DED [11], we contemplated that the application of artificial tears with different HA concentrations may illustrate different efficiency for DED reduction in combination with IPL therapy. This hypothesis was partially supported by the results of the present study.

The patients with DED that received the 0.10% HA artificial tears presented a worse NITBUT recovery after IPL treatment compared to those who received the 0.15% HA artificial tears. In previous research, IPL application showed a significant improvement in both DED signs and symptoms in general DED patients [16]. On the other hand, HA-containing artificial tears reduced the severity of DED more effectively than other types of artificial tears [31]. Nevertheless, the effects of IPL therapy and artificial tears with different HA concentrations on DED management have not been elucidated. To our knowledge, this may be the first study that reports a higher efficiency of DED decline in those that received IPL therapy and high-concentration HA artificial tears. Furthermore, the basic characteristics of the two groups were almost identical; thus, the influence of pre-treatment parameters may be minor. In addition, we adjusted for the effects of age and sex, both known risk factors for DED [1], on the treatment outcomes in the generalized linear model. All the patients who participated in this study live in central Taiwan; thus, the air quality in their residence could be similar. In addition, 90.1% and 92.5% of the patients in the 0.1% and 0.15% HA groups worked in places with air-conditioning; thus, the indoor temperature and relative humidity of their daily conditions may not be significantly different. Furthermore, all the patients used a visual display terminal (including a cellphone, tablet, computer, and television) for at least 7 h per day in their daily life based on their medical records, so the time of digital screen exposure could be very similar among them. Concerning the medications they consumed, no patient used topical non-steroid anti-inflammatory drugs, topical anti-glaucomatous drug, or topical anti-histamine drugs 6 months before their first visit. As a consequence, the application of a high concentration of HA may be an independent indicator for the better post-treatment outcome of IPL regarding NITBUT. The age-based subgroup analysis showed no difference regarding the improvements in DED between the 0.10% and 0.15% HA populations compared to the whole-group analysis, which implies that 0.15% HA may be a universal indicator for better post-treatment NITBUT in all ages. IPL therapy involves the application of heat to the ocular surface and eyelid [14], which can improve the secretion of the meibomian gland and increase tear film stability [32]. Also, HA-containing artificial tears could reduce the ocular inflammatory reaction, which is a critical factor for the stability of tear film [25]. We suppose that the high concentration of HA may have a synergic effect with IPL therapy, thus resulting in a better improvement in NITBUT in the 0.15% HA group. In the previous study, the high-concentration HA reduced interleukin-6 expression in keratinocytes [33], and we speculated that a similar dose-dependent effect of HA may slightly reduce ocular surface inflammation, which could elevate subsequent tear film stability [25]. However, there was no significant difference in the improvement in Schirmer II test results between the two groups. Because the Schirmer II test results are correlated with tear secretion [34], improvements in these results may not be as prominent as those in NITBUT, which is associated with the evaporation and tear film stability that can benefit from IPL therapy and HA. The effectiveness of IPL in managing DED has been illustrated previously [15,16,17]. However, the selection of adjuvant treatments for IPL such as HA-containing artificial tears is also important, which has been demonstrated by the findings of the present study. Still, the correlation cannot be fully confirmed due to the retrospective nature of the present study, in which multiple covariates cannot be controlled well. Further prospective studies should be carried out to evaluate and confirm the causality proposed here.

In terms of the pre-treatment factors predisposing the absence of DED symptom improvement, the multiple pre-treatment DED symptoms correlated with a lower recovery of DED symptoms after treatment in both the 0.10% and 0.15% HA groups. There are two possible explanations for this phenomenon. Firstly, the presence of multiple DED symptoms before treatment may indicate that the patients were more nervous and sensitive; thus, there would be less reduction in the DED-related symptoms post-treatment under such circumstances as a result of psychological factors. In addition, the sensitivity of the corneal nerve plexus in this population may be higher than in the general population, which would lead to permanent and severe DED-related symptoms after refractive surgery [35]. Thus, the high sensitivity of the corneal nerve plexus may lead to the same effect in our study population. Furthermore, a low pre-treatment NITBUT was related to poor symptom recovery in the 0.10% HA group. Because a lower NITBUT is a critical factor for the presence and progression of DED [24], NITBUT may be related to the recovery of a post-treatment symptom in the population that did not receive a highly anti-inflammatory agent (e.g., HA) supplement. On the other hand, only the low pre-treatment NITBUT and Schirmer II test results were associated with no DED sign improvements in either the 0.10% or 0.15% HA groups. There is scant research to demonstrate this result. Both the NITBUT and the Schirmer II test results are critical factors for the development of DED [6]; the low pre-treatment value of these two factors could imply a refractory status and poor outcome regardless of the concentration of HA. However, the worse ocular surface staining did not correlate with poor outcomes, and the exact pathophysiology of this result in the present study needs further evaluation. There were three patients with retinal diseases in the present study and one of them received trans-pars plana vitrectomy. We did not exclude these patients because the retinal surgery was arranged three years before the first visit to our clinic, and all three of the participants/their eyes did not receive additional therapy for the retinal diseases throughout the follow-up interval of our study.

Concerning the effectiveness of IPL therapy in the present study, the tear secretion function improved by about 25 percent after three rounds of IPL therapy and the improvement in NITBUT was around 30 percent. In a preceding study, the tear secretion function was significantly raised after IPL therapy compared to the pre-treatment condition [36], but another study showed a non-significant increment in tear secretion function after IPL therapy [37]. Accordingly, the effects on tear secretion function in the present study may be compatible with earlier publications. Furthermore, the NITBUT demonstrated a significant increment after IPL therapy in the present study, where approximately 25% and 40% of NITBUT augmentation was found in the 0.10% and 0.15% groups after IPL therapy. In a previous study, the NITBUT value was also enhanced after IPL therapy [37], and the present research demonstrated a similar result and efficiency. In addition, the fluorescein corneal stain in the present study was also improved, which is similar to the results in previous studies [16,37]. However, the DED-related symptoms did not improve as significantly as the other objective parameters. DED-related symptoms reduced significantly after IPL therapy in a previous study [16]. Previous publications demonstrated the effectiveness of IPL therapy on DED signs and symptoms [16,36,37], and the addition of HA-containing artificial tears in the present study further elevated the degrees of improvement in NITBUT and Schirmer test results. Still, the extent of improvement in DED-related symptoms in the present study were similar to previous studies. The follow-up period of the present study was not inferior to previous studies [16,36,37]; thus, the mid-term effect of combined IPL and HA-containing artificial tears may be adequate.

There are still some limitations to the present study. Firstly, the retrospective design could have reduced the homogeneity of the two study groups, although no significant difference was illustrated between the groups in all the pre-treatment parameters. Similarly, the post-treatment TMH, eyelid closure rate, and meibomian gland loss rate could not be evaluated since we did not measure these parameters in our routine examination. Also, we did not control for all the environmental and lifestyle factors adequately due to the retrospective nature of the study. These limitations result from the retrospective design and again reduce the integrity of our results. In addition, the usage of 0.10% or 0.15% HA-containing artificial tears was decided by the patients, and this non-randomized selection process could lead to some bias. Moreover, the total number of eyes assessed in the present study was relatively low; only 82 eyes were enrolled into the analysis. Despite the application of non-parametric testing, the statistical power of the present study could be low. Finally, the frequency of HA-containing artificial tear application may be different in daily life for each person, which can affect the analyses to a large extent.

## 5. Conclusions

In conclusion, the usage of 0.15% HA-containing artificial tears resulted in a similar effect on DED signs and symptoms after IPL therapy compared to the 0.10% HA-containing artificial tears. Furthermore, the predisposing factors for minimal DED sign and symptom improvements were also similar for the two concentrations. Consequently, the selection of HA-containing artificial tears may not significantly influence the treatment outcome of IPL therapy. A further large-scale prospective study is mandatory to investigate the effects of HA-containing and other artificial tears on the outcome after IPL therapy.

## Figures and Tables

**Table 1:** The initial characteristics of the two groups.

Feature	0.1% HA Group (N: 42)	0.15% HA Group (N: 40)	*p*
Age (years, mean ± SD)	46.23 ± 11.72	45.96 ± 12.51	0.905
Sex (male: female)	16:26	17:23	0.822
Ocular disease			0.526
Retinal disorder	2	1	
Glaucoma	0	0	
Other	1	0	
Ocular surgery			0.999
Retinal surgery	1	0	
UDVA (LogMAR)	0.38 ± 0.12	0.35 ± 0.13	0.260
Cycloplegia SE (D)	−2.91 ± 1.36	−2.65 ± 1.27	0.342
IOP	12.68 ± 2.74	12.89 ± 3.05	0.713
NITBUT	8.72 ± 3.35	8.84 ± 3.60	0.826
Eyelid closure rate	94.02 ± 5.69	93.78 ± 5.32	0.831
Lipid thickness			0.269
Grade A–C	25	18	
Grade D–E	17	22	
TMH	0.29 ± 0.10	0.28 ± 0.11	0.673
Meibomian gland loss rate	28.59 ± 6.68	29.27 ± 6.31	0.624
Schirmer II test	10.07 ± 4.63	9.84 ± 5.14	0.788
Ocular surface stain			0.651
Grade 0–3	27	23	
Grade 4–6	15	17	
DED-related syndrome			0.729
1	16	18	
2	16	15	
≥3	10	7	

D: diopter; DED: dry eye disease; HA: hyaluronic acid; IOP: intraocular pressure; N: number; SD: standard deviation; SE: spherical equivalent; NITBUT: non-invasive tear break-up time; TMH: tear meniscus height; UDVA: uncorrected distance visual acuity.

**Table 2:** Ophthalmic parameters of the two groups after the intense pulsed light therapy.

Outcome	0.1% HA Group (N: 42)	0.15% HA Group (N: 40)	*p*
UDVA	0.37 ± 0.14	0.35 ± 0.12	0.468
SE	−2.90 ± 1.38	−2.62 ± 1.28	0.313
IOP	13.16 ± 2.48	12.57 ± 3.38	0.334
Schirmer II test	11.74 ± 4.20	12.97 ± 3.46	0.101
NITBUT	10.83 ± 3.14	12.46 ± 3.29	0.023 *
Ocular surface stain			0.788
Grade 0–3	34	31	
Grade 4–6	8	9	
DED-related syndrome			0.508
1	24	25	
2	10	11	
≥3	8	4	

DED: dry eye disease; HA: hyaluronic acid; IOP: intraocular pressure; N: number; SE: spherical equivalent; UDVA: uncorrected distance visual acuity. * denotes significant difference between groups.

**Table 3:** The trend of dry eye improvements between the two populations.

Outcome (Reference: 0.10% HA Group)	aOR	95% CI		*p*
		Lower	Upper	
Schirmer II test	1.140	0.997	1.257	0.054
NITBUT	1.238	1.014	1.377	0.039 *
Ocular surface stain	0.975	0.930	1.099	0.729
DED-related syndromes	1.005	0.942	1.183	0.636

aOR: adjusted odds ratio; CI: confidence interval; DED: dry eye disease; NITBUT: non-invasive tear break-up time. * denotes significant improvement in 0.15% HA group compared to 0.10% HA group.

## Data Availability

The data are available from the corresponding author upon reasonable request.
